# Temporal stability and correlation of EEG markers and depression questionnaires scores in healthy people

**DOI:** 10.1038/s41598-023-49237-4

**Published:** 2023-12-11

**Authors:** Toomas Põld, Laura Päeske, Hiie Hinrikus, Jaanus Lass, Maie Bachmann

**Affiliations:** 1 Department of Health Technologies, School of Information Technologies, Tallinn University of Technology, 5 Ehitajate Rd, 19086 Tallinn, Estonia; 2 Meliva Unimed Qvalitas Medical Centre, Tallinn, Estonia

**Keywords:** Biomedical engineering, Engineering

## Abstract

Mental disorders, especially depression, have become a rising problem in modern society. The development of methods and markers for the early detection of mental disorders is an actual problem. Psychological questionnaires are the only tools for evaluating the symptoms of mental disorders in clinical practice today. The electroencephalography (EEG) based non-invasive and cost-effective method seems feasible for the early detection of depression in occupational and family medicine centers and personal monitoring. The reliability of the EEG markers in the early detection of depression assumes their high temporal stability and correlation with the scores of depression questionnaires. The study was been performed on 17 healthy people over three years. Two hypotheses have been evaluated in the current study: first, the temporal stability of EEG markers is close to the stability of the scores of depression questionnaires, and second, EEG markers and depression questionnaires’ scores are not correlated in healthy people. The results of the performed study support both hypotheses: the temporal stability of EEG markers is high and close to the stability of depression questionnaires scores and the correlation between the EEG markers and depression questionnaires scores is not detected in healthy people. The results of the current study contribute to the interpretation of results in depression EEG studies and to the feasibility of EEG markers in the detection of depression.

## Introduction

Mental disorders, especially depression, have become a rising problem in modern society. According to a recent report by WHO, close to 15% of the world’s working population is estimated to experience a mental disorder^1^. Globally, an estimated 5% of adults suffer from depression^2^. The estimated high rate of depression worldwide has made depression the second leading cause of disability before COVID-19 in 2019^3^. The early detection of mental disorders contributes to the control of the problem and prevention of further increasing severity of the disorder. Therefore, the development of methods and markers for the early detection of symptoms of mental disorders in otherwise healthy people is an actual problem. The electroencephalography (EEG) based non-invasive and cost-effective method seems feasible for the early detection of depression in occupational and family medicine centers and personal monitoring.

Psychological questionnaires are the only tools for evaluating the symptoms of mental disorders in clinical practice today. The commonly accepted assessment tools for depression are based on questionnaires that quantitatively assess the severity of symptoms^4–7^. The Hamilton Depression Rating Scale (Ham-D) is the most widely used tool for clinician-administered assessment of depression and has been a golden standard for over half a century^4^. Despite that, some criticism about the retest reliability and validity of the HAM-D has been expressed^8^.

EEG has been successfully applied for discrimination between depressive and healthy groups in research studies over decades, but the EEG is not used in clinical practice for the assessment of depression. EEG method provides detection of different features of depression in cortical bioelectrical activity^9,10^. The temporal stability and reliability of EEG depression markers are a prerequisite for applicability.

EEG has been reported to detect features specific to depression at the level of different EEG frequency bands^11–15^. Depression patients have evidenced greater overall beta power in a bilateral anterior region^11^. Depressed males displayed increased overall frontal and parietal alpha power^12^. Posterior alpha has been reported to constitute a reliable biomarker of depression^13^. Depression is accompanied by changes in the EEG spectrum and disturbs the balance between the higher and lower frequency bands related to the increase of higher frequencies that are stronger in the parietal region^14^. The linear markers of spectral asymmetry SASI and relative gamma power have demonstrated an increase in depression over the whole brain and higher discrimination ability between depressive and healthy groups in channels FCZ, C3, CZ, CPZ, C4, P8, and C4^15^.

Depression causes alterations not only in the level of EEG signal but increases also the complexity of the signal reflected by nonlinear temporal dynamics of EEG^15–19^. The scaling exponents in detrended fluctuation analyses (DFA) of depressed patients have relatively higher values in all brain regions compared to healthy controls, with significant differences at F3, C3, T3, T4, and O1 channels^17^. The Higuchi fractal dimension (HFD) has demonstrated higher complexity with depression in the left and right frontal lobes of the brain^18^. The nonlinear markers HFD and DFA provided the best classification accuracy between depression and healthy groups in F7, T3, C3, P3, O1, and O2 channels^19^. The combination of linear and nonlinear markers provides high classification accuracy in the left parietal-temporal lobe in the beta EEG frequency band and fewer EEG channels (FP1, FP2, F3, O2, and T3) on depression detection^16^. The nonlinear markers of temporal dynamics HFD, DFA, and Lempel–Ziv complexity (LZC) have demonstrated an increase in all brain areas and the ability to discriminate between depression and healthy groups in the majority of channels reaching the level of significance F3, FC3, F8, FC, C3, FT8, FC4, CZ, CP3, P3, T8, C4, and CP4^15^.

The experimental results reported by various authors have demonstrated quite a similar trend of the increase of EEG power in alpha, beta, and gamma bands and higher complexity of the signal with depression. The spatial distribution of the effect over the cortex is rather diverse. The stronger effect of depression has been reported in various brain areas from anterior to posterior including frontal, temporal, parietal, and occipital channels only partly overlapping in some studies. The spatial diversity of most sensitive EEG channels can be related to the complex nature of depression including diverse symptoms and corresponding neural correlates in the different brain areas^20^.

The reliability of EEG as a tool for the assessment of depression depends on the stability of the EEG markers. Brain bioelectrical activity and EEG signal simultaneously reflect multiple physiological and emotional processes in an individual. Therefore, the natural temporal variability of EEG signal is very high even in the resting state healthy brain. Despite that, the investigations of EEG reliability performed over decades have demonstrated high stability of the EEG power parameters^21–27^. The stability of EEG theta, alpha, and beta bands’ power over two months has been reported higher than delta band power^25,26^. The temporal stability of EEG power markers has been demonstrated from minutes to twelve years^26,28–30^. Only a few data are available for the stability of non-linear markers^30^.

The reliability of the EEG markers in the detection of depression assumes their correlation with the symptoms of depression. The correlation of EEG with high HAM-D scores in patients with diagnosed depression has been demonstrated in many studies over decades^31–34^. In the evaluation of clinical outcomes of depression treatments, HAM-D scores are found to be negatively correlated with EEG delta and theta and positively with beta waves^32^. A significant correlation is reported between HAM-D and LZC in poststroke depression^33^. Gamma power has been significantly related to the post-treatment change in HAM-D scores^34^. A good correlation between SASI and HAM-D scores has been reported in indoor major depression patients^14^. There is no data about the correlation of the EEG markers with the low HAM-D scores. HAM-D scores higher than zero have been reported not only in depression but also in healthy people related to various psychophysiological factors other than depression^35^. The HAM-D scores higher than 7 have been recommended to estimate depression^35^. The EEG markers reflect specific depression signs in the brain and are significantly different in people with depression and healthy people^11,13,15,17,19^. In healthy people, where depression symptoms are not evident, the correlation between EEG markers and depression questionnaires scores is not expected.

The current study aims to compare the temporal stability of EEG markers with the scores of depression questionnaires and investigate the correlations between them in healthy people. Two hypotheses are formulated:

1. The temporal stability of EEG markers is close to the stability of depression questionnaires.
2. EEG markers and depression questionnaires scores are not correlated in healthy people.

To evaluate the hypotheses, the stability of four selected EEG frequency band power markers, four EEG markers describing signal nonlinear dynamics, and the scores of two depression questionnaires are assessed. EEG theta band power (TBP), alpha band power (ABP), beta band power (BBP), and gamma band power (GBP) are selected as being affected by depression and successfully used for discrimination of depression in earlier studies^11,12,15^. Spectral asymmetry index (SASI) is selected as a linear dynamics marker^14^. Higuchi fractal dimension (HFD), detrended fluctuation analyses (DFA), and Lempel–Ziv complexity (LZC) are selected as nonlinear dynamics markers that have been earlier successfully used for the discrimination of depression EEG^15,17,19^. To minimize the impact of external factors, the markers are calculated based on the eyes closed resting state EEG. The clinician-administered Ham-D [HAM] and the self-administered Emotional State Questionnaire for depression (EST-Q-D) are selected as depression questionnaires^4,36^.

## Materials and methods

### Participants and the study protocol

The volunteers were invited to participate in the study among the employees who underwent regular occupational health examinations at the medical center. The selected participants were healthy according to the self-reported questionnaires as well as to the medical and biochemical examinations performed in the medical center. The participants declared no previous brain injuries or mental disorders and were free of current depression episodes and previous history of depression. All the selected participants were non-smoking and right-handed. The selected participants had higher education and were employed as specialists, engineers, and managers.

In total, 17 participants were included in the study, twelve female and five male individuals. The average age of the group was 43.7 ± 5.2 during the first and 46.5 ± 5.5 during the second session.

The participants passed two sessions of examination. The three-year interval between the sessions was determined by the period of regular occupational health examinations. The protocol was identical in both sessions. First, the participants filled out the self-administered EST-Q-D depression questionnaires at home. Second, a medical doctor interviewed the participants and completed the HAM-D depression questionnaire at the medical center. Third, the resting eyes closed EEG was recorded in the medical center.

The study was conducted following the Declaration of Helsinki and approved by the Tallinn Medical Research Ethics Committee, decision No. 2079 from Sept. 28, 2018. All participants were informed about the aim and procedures of the study and they signed the written informed consent.

### Questionnaires

The 17-item Hamilton Depression Rating Scale was used in structured interviews. The interviews were performed in the Medical Centre between 9 and 10 AM for all subjects. The interview was conducted in a separate office, in a room with normal lighting, without distracting sounds. The test subject and the physician sat at the table, facing each other. The physician read out the questions and the subject answered orally. During the interview, the physician solicits patient reports on a variety of depressive symptoms and uses his clinical expertise to assign each a score for severity. The score of the answer to each question was evaluated and noted on the questionnaire sheet by the doctor. At the end of the interview, the total score was calculated and marked on the sheet by the doctor. The same doctor interviewed all participants.

The participants were asked to fill out the EST-Q-D depression questionnaires at home in the morning on the day of their examination in the Medical Centre. They themselves evaluated the scores for 8 questions and calculated the total score.

### EEG recordings

All EEG recordings were performed before noon. The recordings were performed in an isolated dimly lit room. The participants were relaxed and in a lying position. They were asked to abstain from alcohol for 24 h and coffee for 2 h before recording. To minimize the impact of external factors, eyes closed EEG was recorded. The 19 electrodes EEG was recorded for 7 min. The Cadwell Easy II EEG (Kennewick, WA, USA) EEG device was used for recordings. The electrodes were located according to the international 10–20-electrode position classification system. The signals from 18 channels O1, O2, Pz, P4, P8, C4, T8, P7, P3, C3, Fz, F4, F8, T7, F3, FP2, F7, FP1 were recorded using the electrode Cz as reference. The raw EEG signal was recorded at the sampling frequency of 400 Hz in the frequency band 0.3–70 Hz.

### Preprocessing

The raw EEG signals were filtered using a zero-phase Butterworth band-pass 2–47 Hz filter with an attenuation of 100 dB in the stopband. Next, the signal was segmented into 20.48-s segments. Further, the quality of the segments was visually evaluated by an experienced specialist, and segments with artifacts were removed. Finally, 15 artifacts-free EEG signal segments were selected for further analysis. MATLAB software was used for EEG signal processing. In the calculation of linear and nonlinear markers, the marker’s value was determined as the mean value of the calculations’ results over 15 consecutive artifacts-free nonoverlapping segments.

### EEG linear analysis

The EEG theta (TBP), alpha (ABP), beta (BBP), and gamma (GBP) band power markers describe the power of the signal in the different parts of the EEG spectrum. The power markers were calculated in the frequency domain based on the power spectral density (PSD) of the recorded EEG signal. The PSD was calculated using the Welch averaged periodogram method. Each segment was divided into 50% overlapping sections with a length of 2,048 and windowed by the Hanning window. The marker TBP was calculated by averaging the PSD over the frequencies in a fixed frequency band of 4–7 Hz, the marker ABP in 8–12 Hz, the marker BBP in 13–30 Hz, and the marker GBP in the frequency band 31–47 Hz.

### SASI describes the asymmetry of the EEG spectrum in the frequency domain

The balance of EEG power at the frequencies lower and higher than the spectral maximum was calculated, and the spectral maximum (alpha band) was excluded from analyses^14^ (Hinrikus et al., 2009). First, the spectrum of the EEG segment was calculated using the Welsh averaged periodogram method. Second, the boundary frequencies of the lower and higher frequency bands were determined based on the frequency at the maximum of the spectrum f_m_: lower band from (f_m_-6) Hz to (f_m_-2) Hz and higher band from (f_m_ + 2) Hz to (f_m_ + 43) Hz. Third, the levels of the signal in lower S_L_ and higher S_H_ frequency bands were calculated by averaging PSD over the bands’ frequencies. Finally, the SASI was calculated as

1 $$SASI=\frac{{S}_{L}-{S}_{H}}{{S}_{L}+{S}_{H}}$$

### EEG nonlinear analyses

#### HFD describes the self-similarity of the EEG signal in the time domain

The calculation of HFD was based on original algorithms^37^. A segment *L* of *k* samples selected as a scaling unit *L*(*k*) determines the curve that represents the time series as *L*(*k*) ∼ *k*^−*FD*^ where *FD* is the fractal dimension. The value of fractal dimension FD was calculated according to the following algorithm^37^. From a given time series: *X*(1), *X*(2), *X*(3), …, *X*(*N*) a new series $X_{k}^{m}$ is constructed as:

2 $$ {X_{k}^{m} {:}X\left( m \right),X\left( {m + k} \right),X\left( {m + {\text{2k}}} \right),...,X\left( {m + {\text{int}}\left( {\frac{N - m}{k}} \right) \cdot k} \right),\quad m = {1,2},...,k{.}} $$

The length *L*_m_(*k*) of every curve is calculated according to the formula:

3 $$ {L_{m} \left( k \right) = \frac{{1}}{k}\left[ {\left( {\sum\limits_{{i = {1}}}^{{{\text{int}}\left( {\frac{N - m}{k}} \right)}} {\left| {X\left( {m + ik} \right) - X\left( {m + \left( {i - {1}} \right)k} \right)} \right|} } \right)\frac{{N - {1}}}{{{\text{int}}\left( {\frac{N - m}{k}} \right) \cdot k}}} \right]} $$

The length *L(k)* of the curve for time interval *k* is defined as the average over *k* values of *L*_*m*_(*k*), *m = *1, 2, …, *k * If *L(k)* scales like as *L(k) ~ k*^*−FD*^, the curve has fractal dimension FD, which is calculated according to the following formula:

4 $$ FD = \frac{{n\sum {(x_{k} y_{k} )} - \sum {x_{k} \sum {y_{k} } } }}{{n\sum {(x_{k}^{2} ) - \left( {\sum {x_{k} } } \right)^{2} } }} $$

where *x*_*k*_ = ln(1/*k*), *y*_*k*_ = *ln L*(*k*), *k* = *k*_1_,….,*k*_*max*_, and *n* denotes the number of *k*-values for which the linear regression is calculated (*2* ≤ *n* ≤ *k*_*max*_). In the case of EEG having a sample rate of 400 Hz, the selection of *k*_max_ = 40 is appropriate^15^.

### DFA compares the regularity and randomness of the signal

DFA is calculated in the time domain using the published by Peng et al.^38^ algorithms and applying the adaptation to EEG described by Bachmann et al.^15^. First, the EEG signal segment *x*(*i*), where *i* is the length of the segment ranging from 1 to N (N = 2000), was integrated to generate a new time series *y*(*k*),

5 $$ y(k) = \sum\limits_{i = 1}^{k} {\left[ {x\left( i \right) - \overline{x}} \right]} \,\,\,\,\,\,k = 1,...,N $$

where $\overline{x}$ is the average of the EEG signal *x*(*i*). After that, the new time series *y*(*k*) is divided into n equal windows. Window length started from 8 samples up to 400 samples varying equidistantly on a logarithmic scale (0.02 s up to 1.00 s). In each window n, the least squares line, *y*_*n*_(*k*), is fit to the data *y*(*k*) with the fitting range from 0.1 s (excluding the alpha frequency) to 1.0 s, as the brain often suppresses large fluctuations on longer timescales. Next, the local trend *y*_*n*_(*k*) is subtracted from the data *y*(*k*). The root mean square fluctuation of the demeaned, integrated, and detrended signal segment is calculated as:

6 $$ F\left( n \right) = \sqrt {\frac{1}{N}\sum\limits_{k = 1}^{N} {\left[ {y\left( k \right) - y_{n} \left( k \right)} \right]^{2} } } . $$

Those final steps are repeated for all window sizes giving the average fluctuations as a function of window length. Those fluctuations are expected to increase with the window length. The scaling is present in the case if on a log–log graph of F(n) vs. n appears a linear correlation. The slope of the line, that is the scaling exponent α, relating logF(n) to logn describes the type of scaling.

### LZC describes the variability of the signal in a time domain

The LZC was calculated following the original algorithms published by Lempel and Ziv^39^ and Zhang et al.^40^, adjusted for EEG by Bachmann et al.^15^. First, the EEG signal segments were further epoched (epoch length was chosen 2048 samples). For LZC calculation each epoch is converted into binary sequence *s(n)* as follows:

7 $$ s(n) = \left\{ {\begin{array}{*{20}c} {1,\,\,\,\,\,if\,\,\,\,\,x(n) > m} \\ {0,\,\,\,\,if\,\,\,\,\,x(n) \le m} \\ \end{array} } \right., $$

where x(n) is the epoch, n is the epoch’s sample index from 1 to *N* (epoch length) and m is the threshold value. Thereafter, the resulting binary sequence s(n) is scanned from left to right counting the number of different patterns occurring. The complexity value c(n) is increased every time a new pattern is encountered. The upper bound of c(n) is

8 $$ \mathop {\lim }\limits_{n \to \infty } c(n) = b(N) = \frac{N}{{\log_{a} N}} $$

where a is the number of different patterns, and *N* is the epoch’s length. To avoid the variations due to the epoch length, normalized LZC values are calculated as follows:

9 $$ C(N) = \frac{c(N)}{{b(N)}} $$

### Statistics

The EEG markers were calculated for each subject in all 18 EEG channels. Whereas the most sensitive channels vary in different studies^11–19^, the average value of a marker over the whole cortex was selected for statistical evaluation. The Wilcoxon Signed-Rank Test was used for the statistical evaluation of EEG signal level between two sessions. The comparisons of eight EEG markers were performed in the same EEG database. The Bonferroni corrected for multiple comparisons confidence interval p < 0.05/8 = 0.00625 was considered statistically significant. The HAM-D and EST-Q-D questionnaires are independent of EEG but contain similar questions in both. The significance level for the questionnaires is 0.05/2 = 0.025.

The alterations in signal level between the two sessions were small. Therefore, the linearity of correlation between the markers in two sessions was presumed and Pearson’s correlation coefficient was used for the calculation of correlations. The number of correlation tests between a score and 8 EEG markers n = (8 × 8–8)/2 = 28. The Bonferroni correction determines the confidence level for correlation coefficients p < 0.05/28 = 0.0018. The significance of the correlation coefficient between the two sessions was assessed using Student’s t-test.

## Results

Table 1 presents the values of averaged over 18 channels EEG markers and depression questionnaires scores in the first and the second session and their standard deviations. The levels of standard deviations are high and partly comparable with the average values of the EEG markers and questionnaires scores. The differences between the values of markers and scores between the two sessions are not statistically significant. The EEG markers in all 18 individual channels are presented in Table S1 in the Supplement. The EEG markers behave similarly in all individual channels: the values of markers in two sessions are correlated and p-values between the sessions are higher than the level of significance. The standard deviations of markers in individual channels are comparable to or somewhat higher than these for the averaged over all channels values in Table 1.

**Table 1:** The average values and standard deviations of EEG markers and depression scores (*n* = 17) in two sessions, and the Wilcoxon signed-rank test *p* values between the sessions calculated for the averaged over all 18 EEG channels signals.

Marker	Average		St dev		St dev/av %		*P*
	1	2	1	2	1	2	
TBP	361	313	274	196	75.9	62.4	0.29
ABP	1551	1334	1972	1689	127	126	0.23
BBP	601	530	270	261	59.5	49.3	0.33
GBPl	75	74	47	53	62.7	70.8	0.78
SASI	0.22	0.23	0.25	0.24	116	99.4	0.38
HFD	1.078	1.079	0.026	0.028	3.3	3.1	0.76
DFA	0.370	0.368	0.087	0.097	23.6	26.4	0.98
LZC	0.353	0.356	0.036	0.035	10.2	9.79	0.54
HAM-D	10.06	7.18	6.76	5.75	67.2	80.2	0.31
EST-Q-D	10.47	7.18	7.41	5.88	71.2	82	0.04

The p < 0.00625 for EEG markers and p < 0.025 for depression questionnaires’ scores indicate statistical significance.

**Figure 1:**
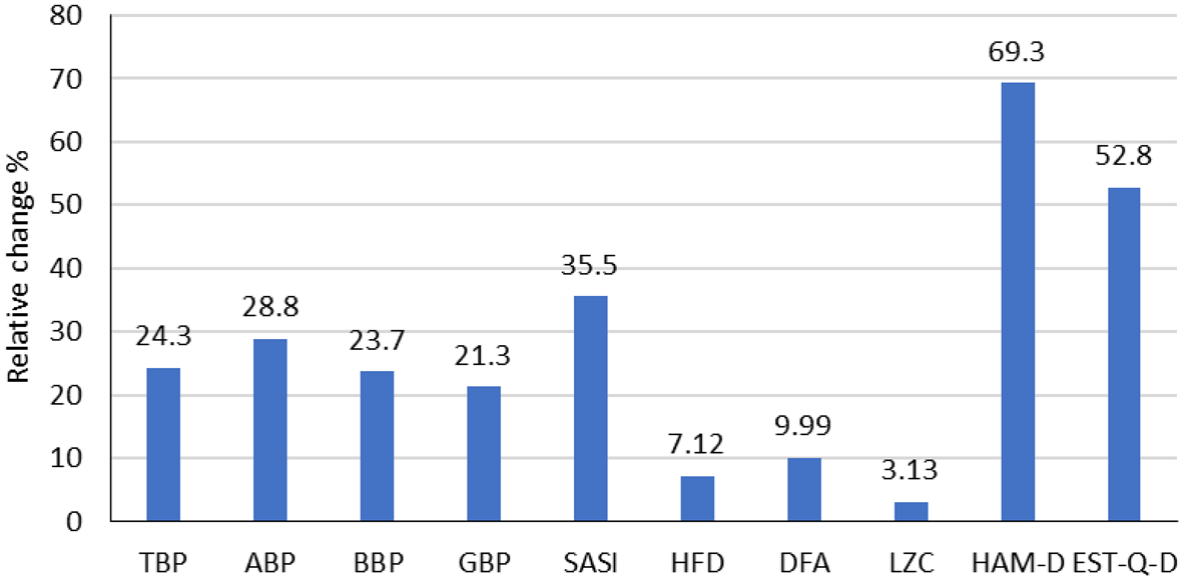
Relative changes in the values of individual EEG markers and questionnaires’ scores between the first and second sessions calculated as averaged over all 17 participants absolute values of individual changes RC $=\frac{1}{17}{\sum }_{k=1}^{17}\left|\frac{{M}_{1k}-{M}_{2k}}{{M}_{1k}}\right|$, where M_1k_ is the value of the marker of a participant k in the first and M_2k_ in the second session.

Figure 1 presents averaged relative changes between two sessions in markers and depression scores. The relative change RC_M_ of a marker M between two sessions was calculated by averaging absolute values of differences between individual markers in the first M_ik_ and the second M_2k_ sessions over the group as RC_M_ $=\frac{1}{17}{\sum }_{k=1}^{17}\left|\frac{{M}_{1k}-{M}_{2k}}{{M}_{1k}}\right|.$ The average relative changes between the EEG markers are limited to 35.5%, lower than the relative change of depression questionnaires scores limited to 69.3%.

**Figure 2:**
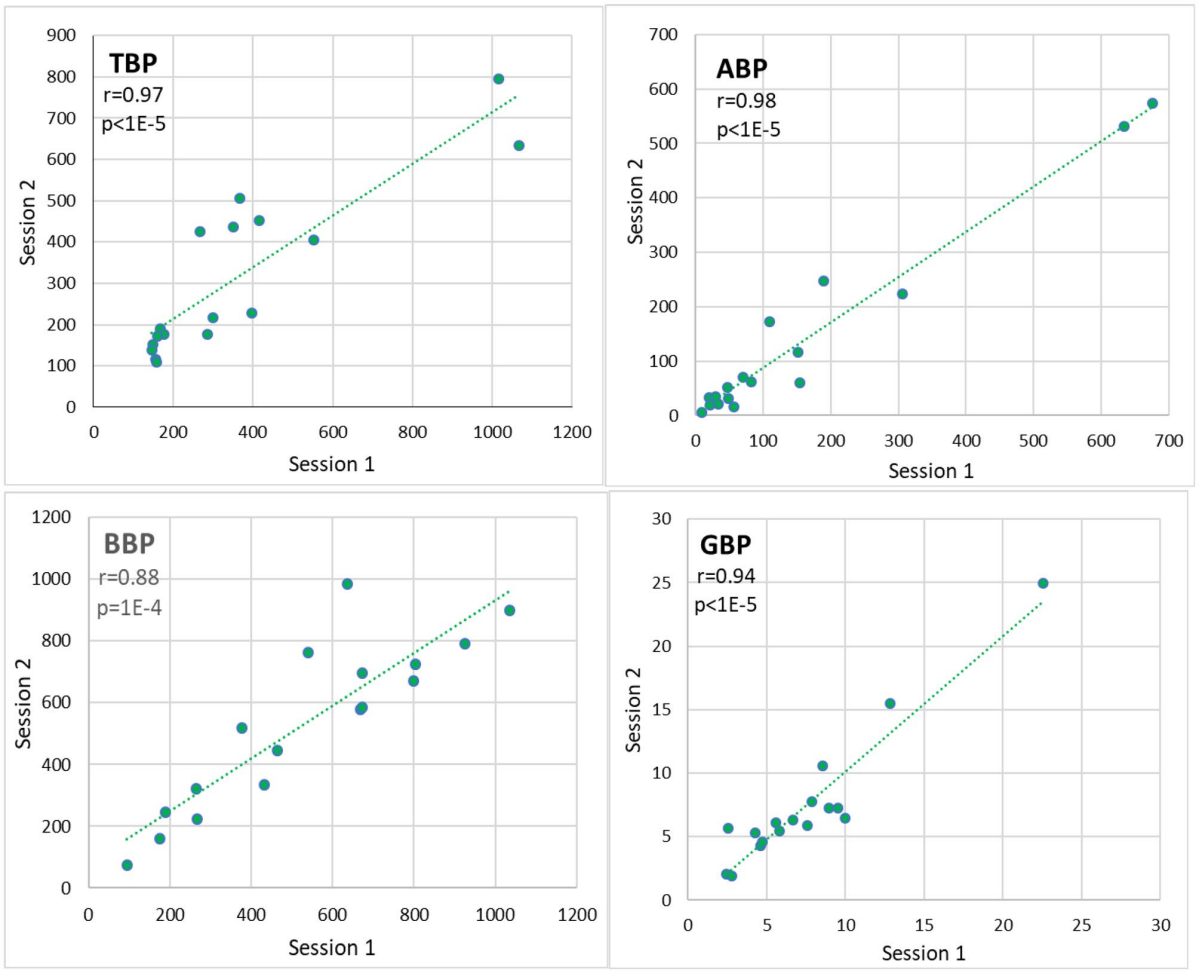
The correlation of EEG power markers theta band power (TBP), alpha band power (ABP), beta band power (BBP), and gamma band power (GBP) between two sessions. The calculated Pearson’s correlation coefficients *r* between two sessions and corresponding *p*-values are indicated (*n* = 17). The *p* < 0.0018 indicates statistical significance.

Figure 2 presents the correlation of EEG power markers TBP, ABP, BBP, and GBP between two sessions. The correlation coefficients between the two sessions are high |*r*|> 0.88 and the calculated *p* < 1E−4 indicate statistical significance of correlation for all EEG power markers.

**Figure 3:**
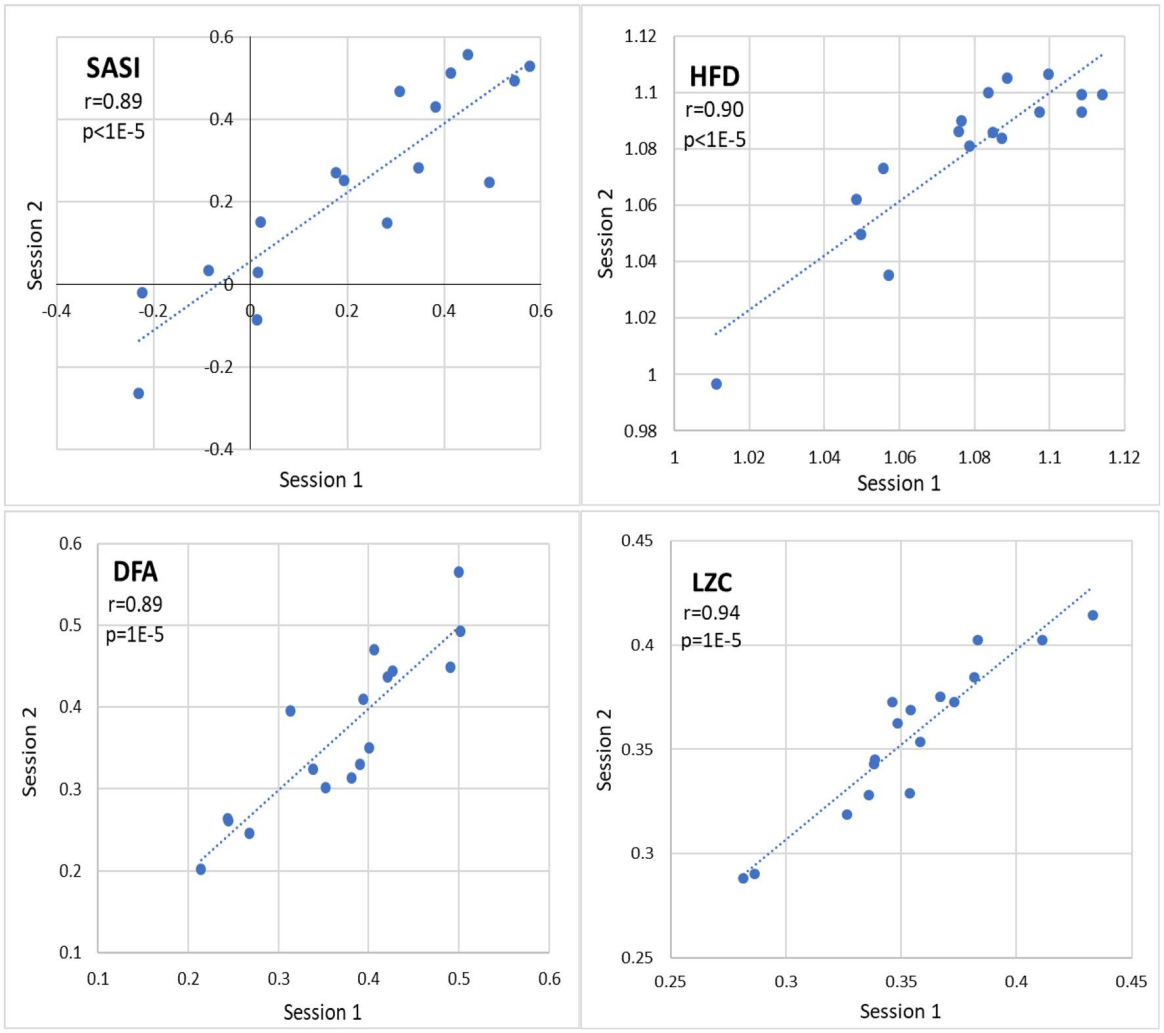
The correlation of EEG dynamics markers spectral asymmetry index (SASI), Higuchi’s fractal dimension (HFD), detrended fluctuations analyses (DFA), and Lempel–Ziv complexity (LZC) between two sessions. The calculated Pearson’s correlation coefficients r between two sessions and corresponding *p*-values are indicated (*n* = 17). The *p* < 0.0018 indicates statistical significance.

Figure 3 presents the correlation of EEG dynamics markers SASI, HFD, DFA, and LZC between two sessions. The correlations between all dynamic markers are high and the correlation coefficients between two sessions |*r*|> 0.89 and the calculated *p* < 1E−5 indicate statistically significant correlations.

**Figure 4:**
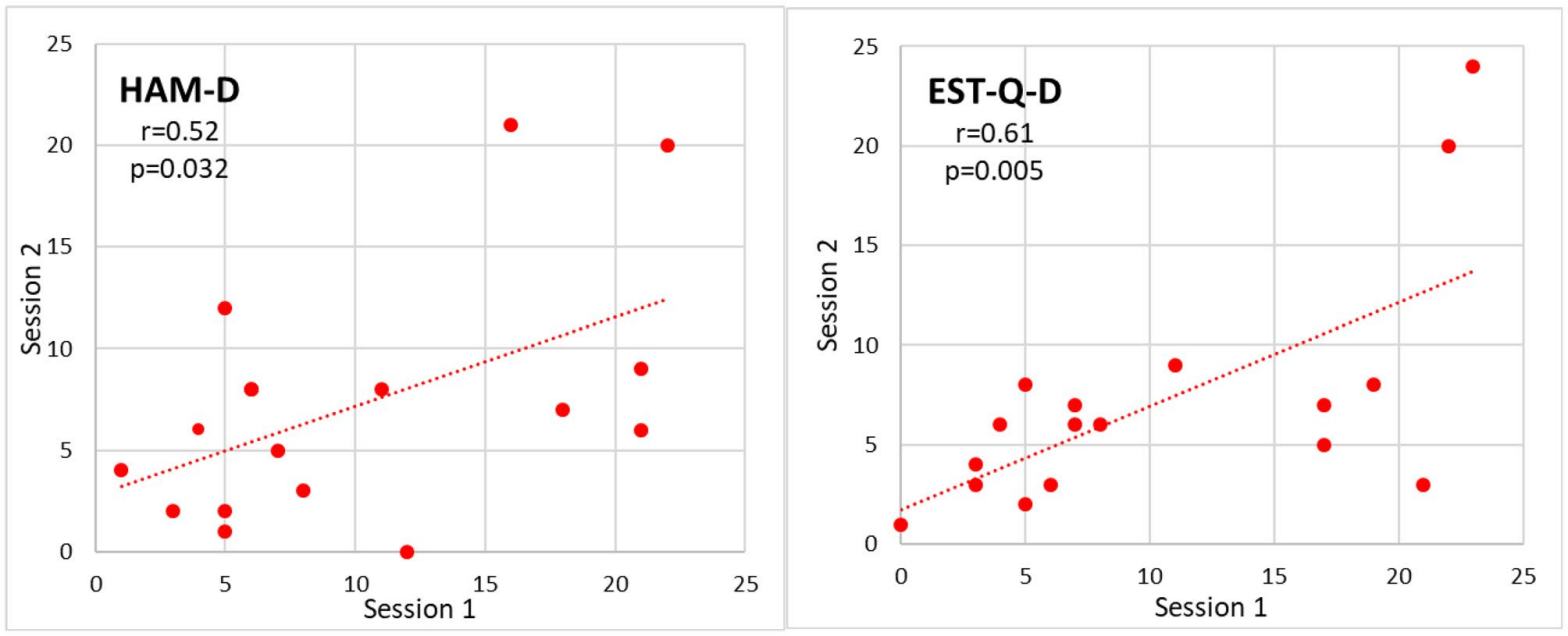
The correlation of depression questionnaires HAM-D and EST-Q-D scores between two sessions. The calculated Pearson’s correlation coefficients r between two sessions and corresponding *p*-values are indicated (*n* = 17). The *p* < 0.025 indicates statistical significance.

Figure 4 shows the correlation of depression questionnaires HAM-D and EST-Q-D scores between two sessions. The correlation coefficients |*r*|= 0.52 and the calculated p = 0.032 of HAM-D and |*r*|= 0.61 and p = 0.005 of ESTQ-D indicate a statistically significant correlation between the two sessions.

**Figure 5:**
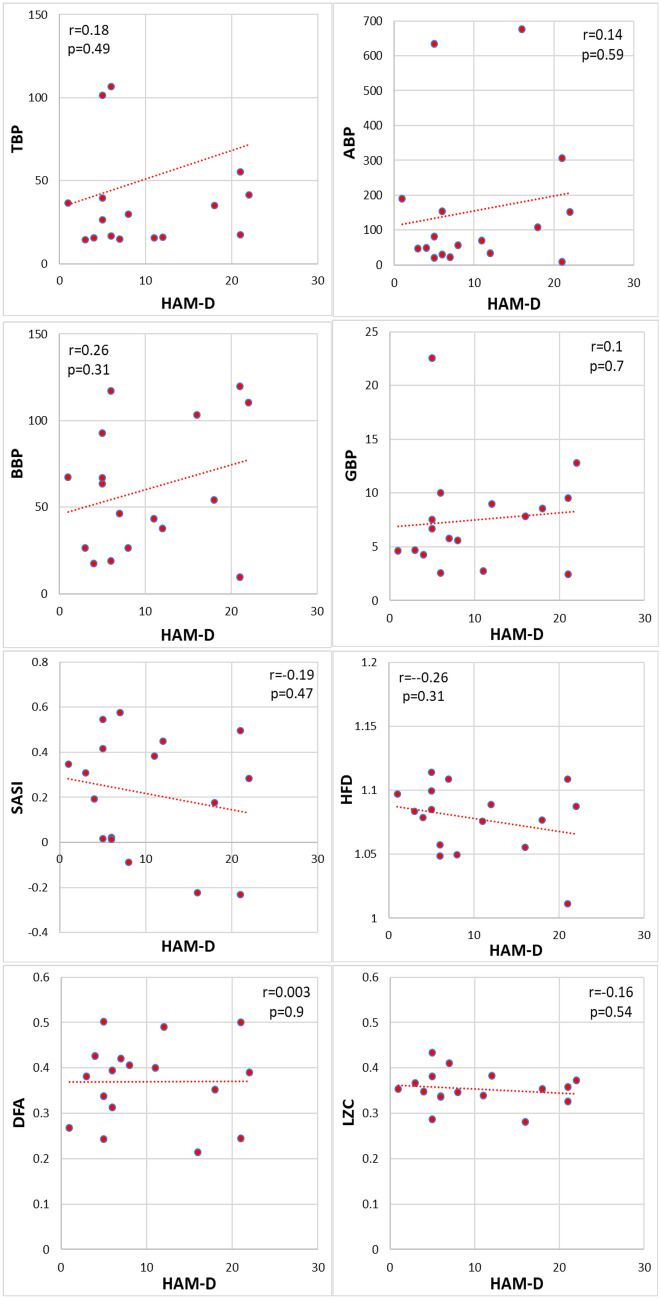
The correlation of HAM-D scores with EEG markers. The calculated Pearson’s correlation coefficients r between two sessions and corresponding *p*-values are indicated (*n* = 17). The *p* < 0.0018 indicates statistical significance.

Figure 5 presents the correlation between the scores of HAM-D and EEG markers. The correlation coefficients of HAM-D scores with different EEG markers |*r*|< 0.26 and the calculated p > 0.31 indicate a significant correlation with none of the EEG markers.

**Figure 6:**
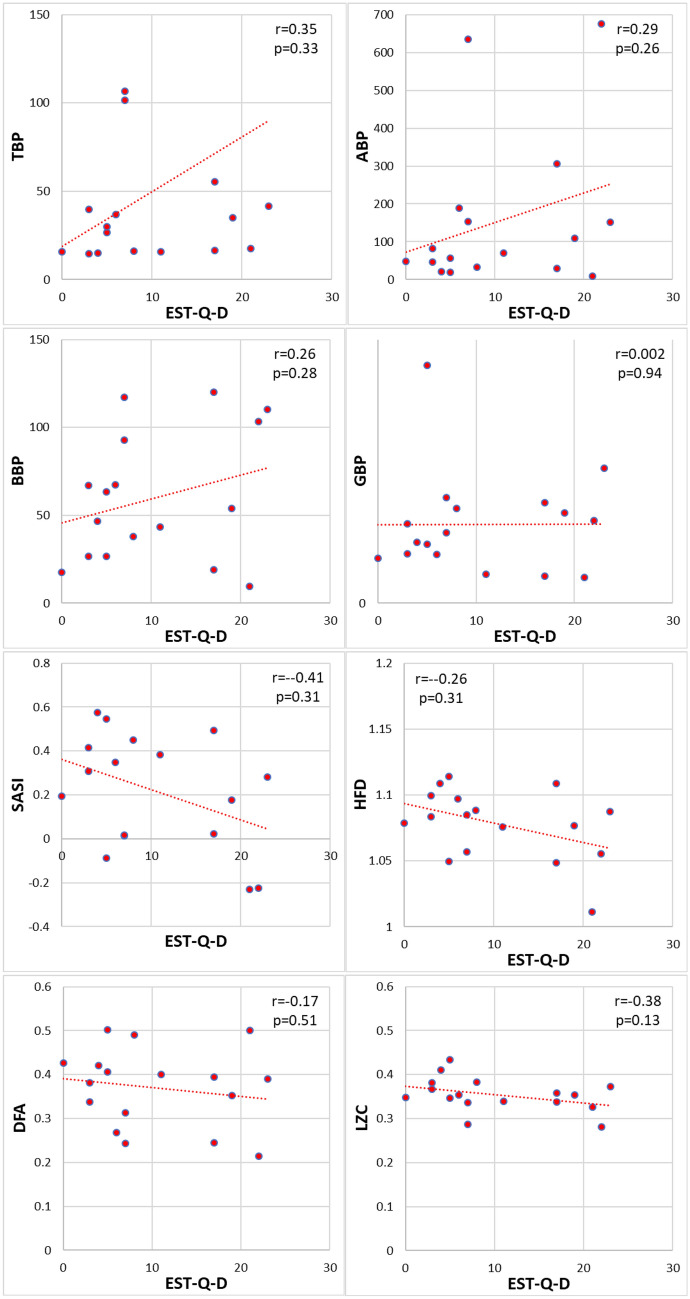
The correlation of EST-Q-D scores with EEG markers. The calculated Pearson’s correlation coefficients r between two sessions and corresponding *p*-values are indicated (*n* = 17). The *p* < 0.0018 indicates statistical significance.

Figure 6 presents the correlation between the scores of EST-Q-D and EEG markers. The correlation coefficients of EST-Q-D scores |*r*|< 0.41 and p > 0.13 indicate no significant correlation with any of the EEG markers.

For comparison of the correlations between the scores of depression questionnaires and EEG markers in Figs. 5 and 6, the correlation coefficients between the EEG markers and questionnaires scores themselves are calculated. The correlation calculated between the scores of two depression questionnaires HAM-D and EST-Q-D |*r*|= 0.85 is statistically significant (*p* = 1.6E−5). Table 2 presents the correlation coefficients between the EEG markers. The correlation is statistically significant between several EEG markers ABP and TBP *r* = 0.84, ABP and BBP *r* = 0.64, HFD and SASI *r* = 0.89, HFD and LZC *r* = 0.69, DFA and TBP *r* = −0.66, DFA and ABP *r* = −0.81, and DFA and BBP *r* = −0.69.

**Table 2:** Pearson correlation coefficients between EEG markers.

Marker		TBP	ABP	BBP	GBP	SASI	HFD	DFA	LZC
TBP	*r*		**0.84**	0.59	−0.077	−0.50	−0.18	−**0.66**	−0.39
	*p*		**2.5E**−**5**	0.013	0.768	0.041	0.489	**1.3E**−**3**	0.122
ABP	*r*	**0.84**		**0.64**	−0.113	−0.37	0.01	−**0.81**	−0.61
	*p*	**2.5E−5**		**1.6E−3**	0.674	0.144	0.970	**8E-5**	0.009
BBP	*r*	0.59	**0.64**		−0.047	0.08	0.35	−**0.69**	−0.17
	*p*	0.013	**1.6E−3**		0.879	0.760	0.168	**1E**−**3**	0.514
GBP	*r*	−0.087	**−**0.113	**−**0.047		0.16	0.25	0.22	0.11
	*p*	0.768	0.674	0.879		0.539	0.333	0.396	0.674
SASI	*r*	−0.50	**−**0.37	0.08	0.16		**0.89**	0.20	0.28
	*p*	0.041	0.144	0.760	0.539		**1E-5**	0.441	0.276
HFD	*r*	−0.18	0.01	0.35	0.25	**0.89**		−0.14	**0.69**
	*p*	0.489	0.970	0.168	0.333	**1E**−**5**		0.592	**1E**−**3**
DFA	*r*	**−0.66**	**−0.81**	**−0.69**	0.22	0.20	−0.14		0.28
	*p*	**1.3E−3**	**8E−5**	**1E**−**3**	0.396	0.441	0.592		0.276
LZC	*r*	**−**0.39	**−**0.61	−0.17	0.11	0.28	**0.69**	0.28	
	*p*	0.122	0.009	0.514	0.674	0.276	**1E**−**3**	0.276	

The values p < 0.0018 indicate the statistical significance of correlation (marked in bold).

## Discussion

### Temporal stability of the EEG markers and the scores of depression questionnaires (the first hypothesis)

The results presented in Table 1 support the first hypothesis is that the temporal stability of EEG markers is close to the stability of depression questionnaires scores. The Wilcoxon Signed-Rank Test *p* values higher than 0.23 for the averaged over all channels values of the EEG markers show no statistically significant differences between the data in the two sessions (Table 1). The *p*-values 0.31 for HAM-D and p = 0.04 for EST-Q-D test scores between the two sessions (Table 1) indicate no statistical significance. The average values of the HAM-D scores of 10 ± 6.76 (first session) and 7 ± 5.75 (second session) are consistent with the results that 84% of healthy controls scored 7 or less on the HAM-D, and 97.5% scored 10 or less^35^. The standard deviations are very high for the EEG power markers and questionnaires scores, and somewhat lower for EEG dynamics markers (Table 1). The standard deviations are comparable or even higher (ABP, SASI) than the average values of the marker or score. The high values of standard deviations of markers reflect a large difference between the markers of individual participants.

The results presented in Fig. 1 also do not reject the first hypothesis. The relative changes in the level of EEG markers between two sessions strongly vary for different markers. The relative changes in the levels of the EEG power markers are limited to 27.8% (ABP) and the EEG dynamics markers to 35.5% (SASI) (Fig. 1). The nonlinear dynamics time domain markers, HFD, DFA, and LZC, indicate the lowest change. The relative change in depression questionnaires’ scores between two sessions (52.8–69.3%) is much higher than in the EEG markers (3.13–35.5%) (Fig. 1). Whereas the questionnaires’ scores between the two sessions do not differ significantly (Table 1), the higher relative change in the questionnaires scores compared to EEG markers is not a sufficient reason to reject the first hypothesis. Such a result may be due to large interindividual variability in a relatively small group.

The results of correlations of EEG markers and depression questionnaires’ scores between two sessions presented in Figs. 2, 3, and 4 support the first hypothesis of similarity of temporal stability between EEG markers and depression questionnaires’ scores. The Pearson correlation coefficients of EEG power markers between two sessions r > 0.88 indicate a statistically significant correlation between the data in two sessions (Fig. 2). The correlation coefficients r > 0.89 indicate a statistically significant correlation of the EEG dynamic markers between two sessions (Fig. 3). These results are consistent with previous results reported by other authors where high reliability of EEG markers has been demonstrated^25,26,29,30^. The correlation r = 0.98 for alpha power over three years in the current study is consistent with the Pearson correlation r = 0.848 of alpha over 12 years^29^. The good reliability of theta, alpha, and beta power has been reported over two months with the reported intraclass correlation coefficient ICC > 0.67 (n = 15), but the reliability of delta and gamma power is somewhat less ICC > 0.41^25^.

The correlation coefficient of HAM-D r = 0.52 and EST-Q-D r = 0.61 (Fig. 4) indicate the statistically significant correlations of the scores between the two sessions. The correlation of depression questionnaires between the sessions (Fig. 4) is lower than the correlation of EEG markers (Figs. 2 and 3). The test–retest reliability of HAM-D over days has been reported to range between 0.65 and 0.98 and is generally decreasing with extending the interval between two measurements^41^. The lower correlation in the current study is related to a much longer time between the sessions, three years.

### Correlation between EEG markers and the scores of depression questionnaires (the second hypothesis)

The results presented in Figs. 5 and 6 support the second hypothesis that the EEG depression markers are not correlated with the scores of depression questionnaires. The values of the correlation coefficient of HAM-D are lower r < 0.26 (p > 0.31) and of EST-Q-D r < 0.41 (p > 0.13) indicating no statistical significance of the correlation between the HAM-D and EST-Q-D with any of the discussed EEG markers. The results do not confirm a correlation between EEG markers and depression questionnaires scores in healthy people.

This result does not contradict the existing knowledge that EEG is correlated with the severity of depression and HAM-D scores in patients with diagnosed depression^31–34^. The character of correlation between EEG markers and questionnaires scores differs at high scores where the clinical symptoms of depression are evident and at low scores in healthy people where no symptoms are evident despite the non-zero scores caused by various factors other than depression. EEG markers reflect characteristic features of depression in the brain and differ significantly between the depression and healthy people^11,13,15,17,19^. In healthy people, the depression symptoms are not evident and the related features in the brain are missing.

Figure 4 (and Table 1) indicates a remarkable trend of decrease of HAM-D and EST-Q-D scores in session 2 which is much less for EEG power markers in Fig. 2 and not noticeable for EEG dynamics markers in Fig. 3. This can be explained by the fact that when passing the evaluation a second time, the procedure and questions already seem familiar to the participants, and the previous experience and a learning effect would affect the results. The learning effect is much stronger in HAM-D and EST-Q-D scores than in EEG markers.

The results demonstrate a correlation between the scores of HAM-D and EST-Q-D questionnaires. The correlation between the scores of two different questionnaires supports their reliability. The character of the correlation between various EEG markers (Table 2) is consistent with the results of a previous study performed in a much larger sample size (n = 80)^42^. The correlation is evident between the ABP and next to alpha band theta and beta band markers TBP and BBP. GBP is not correlated with other markers. HFD is correlated with SASI and LZC. DFA has a negative correlation with power markers TBP, ABP, and BBP. Such a coincidence of results achieved in two different databases supports the quality of the evaluation.

The results of the study contribute to the interpretation of results in depression EEG studies and the feasibility of EEG markers in the detection of depression. The perspective of the applicability of EEG markers for early detection of depression needs further much larger-scale investigations.

### Limitations of the study

The main limitation is the relatively small number of participants. A much larger number of people completed the first session but were no more available for the second session A much larger number of people completed only the first session but were no more available for the second session due to various reasons. Due to the small number of participants, the current study can be considered as a preliminary. Future investigations involving larger numbers of participants are recommended.

The second limitation is the questionable use of depression questionnaires for this purpose. HAM-D is applicable at an individual level. However, the HAM-D was designed to be used as a clinical interview with a depressed patient to measure treatment outcomes and is not designed to evaluate symptoms of depression in healthy persons^4,5^. Despite that, the HAM-D has been successfully used in healthy controls in many studies^35^. The EST-Q-D has been designed to distinguish between different groups of the population and validate discriminating groups of healthy persons and patients. The questionnaire is not validated for differentiation at an individual level^36^. In the current study, mental health is a part of the occupational health inspection survey package and the HAM-D and EST-Q-D have been used to clarify whether further examination by a psychiatrist is an option.

The third limitation is the possibility that some factors (family problems, diseases, etc.) would affect participants' well-being and EEG between two sessions. To minimize the impact of strong extraneous factors, the participants were interviewed before the second session and some persons were excluded.

### Supplementary Information

Supplementary Table S1.

## Data Availability

Data are available upon request from the corresponding author.
