# Supplementary material for: Temporal stability and correlation of EEG markers and depression questionnaires scores in healthy people
Source: Sci Rep. 2023 Dec 11;13:21996. doi: 10.1038/s41598-023-49237-4 (PMC10713782; doi:10.1038/s41598-023-49237-4)
Supplement: Supplementary file 1 — Supplementary Table S1. [file 41598_2023_49237_MOESM1_ESM.pdf]

Supplement 1

Table S1. The average values AV1 and AV2, standard deviations SD1 and SD2, relative standard deviations RSD1 % and RSD2 % of EEG markers in two sessions, and the Wilcoxon Signed-Rank Test *p* values and the Pearson correlation coefficients *r* between the sessions in 18 EEG channels (*n*=17). The p<0.00625/18=0.000347 indicates statistical significance.

**TBP**																		
	**O2**	**O1**	**PZ**	**P4**	**P8**	**C4**	**T8**	**P7**	**P3**	**C3**	**FZ**	**F4**	**F8**	**T7**	**F3**	**FP2**	**F7**	**FP1**
AV1	15.036	13.659	3.907	6.374	12.244	2.502	9.920	11.981	5.671	2.188	3.547	4.855	10.723	10.059	4.525	10.353	10.898	10.091
SD1	20.405	18.837	5.597	8.725	15.825	2.919	14.375	17.129	7.244	2.273	4.750	6.449	13.785	12.766	5.550	13.197	13.601	12.720
AV2	10.856	9.812	3.182	4.276	8.997	2.000	7.516	9.896	4.570	2.234	2.838	3.791	8.847	8.625	4.032	8.453	9.918	8.286
SD2	11.946	9.963	2.725	3.704	10.181	1.807	8.313	11.532	4.288	2.114	2.772	3.408	8.549	10.393	3.928	7.197	10.105	7.179
RSD1 %	135.704	137.905	143.27	136.88	129.244	116.689	144.912	142.962	127.733	103.889	133.90	132.83	128.55	126.90	122.65	127.48	124.80	126.04
RSD2 %	110.04	101.54	85.659	86.637	113.15	90.323	110.61	116.53	93.849	94.590	97.702	89.902	96.628	120.49	97.167	85.138	101.89	86.637
p	0.107	0.142	0.379	0.141	0.073	0.176	0.146	0.181	0.194	0.794	0.230	0.208	0.267	0.117	0.650	0.240	0.440	0.220
r	0.937	0.929	0.912	0.907	0.948	0.915	0.978	0.984	0.960	0.948	0.940	0.956	0.925	0.973	0.971	0.967	0.949	0.970
**ABP**																		
	**O2**	**O1**	**PZ**	**P4**	**P8**	**C4**	**T8**	**P7**	**P3**	**C3**	**FZ**	**F4**	**F8**	**T7**	**F3**	**FP2**	**F7**	**FP1**
AV1	81.232	67.784	17.709	30.464	49.480	6.884	20.753	39.900	23.079	5.049	6.644	8.237	15.813	21.008	8.536	14.764	17.157	15.176
SD1	108.87	85.651	24.682	44.782	68.239	9.415	29.532	49.911	29.467	6.871	11.603	13.021	22.973	30.748	14.958	22.495	25.909	23.906
AV2	76.510	50.109	17.740	22.543	33.858	4.969	16.157	34.301	21.631	4.678	7.122	8.621	14.808	19.655	8.347	14.263	16.945	14.792
SD2	106.19	63.799	22.770	29.240	46.782	7.053	22.649	44.269	27.027	5.420	12.575	14.452	21.724	29.235	13.074	21.697	25.014	22.404
RSD1 %	134.03	126.35	139.37	146.99	137.91	136.75	142.29	125.08	127.67	136.08	174.63	158.08	145.28	146.36	175.23	152.36	151.01	157.52
RSD2 %	138.79	127.31	128.35	129.70	138.16	141.94	140.17	129.06	124.94	115.87	176.57	167.62	146.70	148.74	156.63	152.12	147.62	151.45
p	0.616	0.106	0.984	0.119	0.026	0.139	0.051	0.061	0.379	0.681	0.417	0.631	0.457	0.416	0.760	0.680	0.871	0.732
r	0.938	0.878	0.967	0.942	0.964	0.848	0.975	0.977	0.976	0.849	0.984	0.978	0.972	0.976	0.978	0.976	0.979	0.983

**BBP**																		
	**O2**	**O1**	**PZ**	**P4**	**P8**	**C4**	**T8**	**P7**	**P3**	**C3**	**FZ**	**F4**	**F8**	**T7**	**F3**	**FP2**	**F7**	**FP1**
AV1	20.669	19.772	5.584	10.462	16.173	4.859	10.957	14.739	8.990	4.079	3.311	5.340	8.925	10.363	4.665	8.497	8.855	8.114
SD1	15.678	14.489	4.104	7.607	10.307	3.026	6.194	9.574	5.850	2.426	2.486	3.641	5.157	6.071	3.038	5.124	5.033	4.944
AV2	17.422	14.579	5.531	8.367	12.912	3.924	9.439	13.560	8.282	3.941	3.046	4.658	8.673	11.191	4.617	7.671	8.538	7.475
SD2	12.662	8.698	3.070	4.995	7.573	2.512	4.580	7.209	4.350	1.990	1.873	2.893	5.257	5.865	3.582	4.156	4.349	3.680
RSD1 %	75.852	73.282	73.489	72.712	63.733	62.270	56.532	64.954	65.073	59.466	75.093	68.185	57.782	58.579	65.120	60.298	56.833	60.936
RSD2 %	72.678	59.659	55.510	59.702	58.649	64.028	48.520	53.162	52.528	50.510	61.494	62.114	60.617	52.405	60.929	54.176	50.941	49.232
p	0.218	0.105	0.924	0.147	0.067	0.126	0.172	0.304	0.424	0.753	0.533	0.297	0.828	0.554	0.623	0.427	0.690	0.490
r	0.748	0.519	0.841	0.666	0.749	0.642	0.709	0.889	0.796	0.692	0.725	0.703	0.591	0.552	0.758	0.612	0.773	0.662
**GBP**																		
	O2	O1	PZ	P4	P8	C4	T8	P7	P3	C3	FZ	F4	F8	T7	F3	FP2	F7	FP1
AV1	1.341	1.288	0.471	0.753	1.432	0.504	2.055	1.407	0.729	0.476	0.488	1.194	1.673	1.730	0.793	1.937	1.696	1.755
SD1	0.857	0.813	0.398	0.518	1.113	0.371	1.866	1.256	0.536	0.334	0.429	1.404	1.310	1.176	0.708	1.195	1.285	0.952
AV2	1.329	1.112	0.497	0.679	1.193	0.531	1.932	1.311	0.679	0.479	0.447	0.901	1.725	3.114	0.796	1.642	1.689	1.669
SD2	0.879	0.799	0.409	0.582	0.968	0.548	1.689	1.161	0.583	0.436	0.459	1.163	1.547	3.800	0.924	1.255	1.477	1.157
RSD1 %	63.921	63.109	84.504	68.731	77.701	73.633	90.802	89.301	73.492	70.190	87.933	117.54	78.262	67.978	89.336	61.686	75.734	54.247
RSD2 %	66.147	71.840	82.420	85.695	81.134	103.23	87.421	88.520	85.778	90.990	102.80	129.11	89.657	122.02	116.00	76.444	87.411	69.322
p	0.884	0.031	0.652	0.260	0.005	0.709	0.698	0.113	0.269	0.936	0.268	0.180	0.678	0.105	0.381	0.073	0.961	0.538
r	0.928	0.928	0.841	0.891	0.967	0.861	0.743	0.984	0.952	0.912	0.945	0.790	0.950	0.537	0.980	0.866	0.925	0.874
**SASI**																		
	**O2**	**O1**	**PZ**	**P4**	**P8**	**C4**	**T8**	**P7**	**P3**	**C3**	**FZ**	**F4**	**F8**	**T7**	**F3**	**FP2**	**F7**	**FP1**
AV1	0.201	0.222	0.231	0.308	0.260	0.366	0.243	0.260	0.311	0.359	0.111	0.194	0.074	0.205	0.154	0.086	0.061	0.068

SD1	0.280	0.283	0.284	0.275	0.291	0.304	0.317	0.299	0.289	0.282	0.338	0.332	0.291	0.260	0.316	0.278	0.275	0.265
AV2	0.292	0.257	0.250	0.349	0.298	0.369	0.267	0.300	0.337	0.341	0.125	0.187	0.103	0.281	0.187	0.065	0.085	0.076
SD2	0.259	0.260	0.275	0.271	0.274	0.264	0.272	0.260	0.284	0.287	0.322	0.312	0.254	0.272	0.266	0.312	0.206	0.296
RSD1 %	139.71	127.44	122.92	89.334	111.70	83.188	130.27	115.20	92.735	78.596	304.19	170.95	393.77	126.78	205.49	322.73	449.81	387.79
RSD2 %	88.473	101.29	109.88	77.695	91.831	71.427	101.86	86.672	84.102	84.226	256.48	167.44	247.02	97.017	142.18	477.30	242.85	388.51
p	0.033	0.394	0.683	0.261	0.178	0.921	0.546	0.240	0.511	0.627	0.718	0.847	0.441	0.098	0.212	0.647	0.562	0.861
r	0.824	0.825	0.762	0.863	0.924	0.881	0.870	0.890	0.847	0.868	0.882	0.878	0.854	0.779	0.877	0.813	0.804	0.795
**HFD**																		
	**O2**	**O1**	**PZ**	**P4**	**P8**	**C4**	**T8**	**P7**	**P3**	**C3**	**FZ**	**F4**	**F8**	**T7**	**F3**	**FP2**	**F7**	**FP1**
AV1	1.713	1.713	1.715	1.721	1.715	1.727	1.708	1.713	1.721	1.726	1.680	1.697	1.659	1.699	1.686	1.656	1.654	1.655
SD1	0.045	0.044	0.048	0.045	0.042	0.049	0.061	0.046	0.050	0.046	0.059	0.061	0.050	0.044	0.056	0.052	0.051	0.051
AV2	1.719	1.715	1.713	1.726	1.714	1.731	1.711	1.717	1.723	1.720	1.682	1.693	1.665	1.715	1.691	1.656	1.659	1.661
SD2	0.053	0.045	0.059	0.049	0.042	0.046	0.055	0.040	0.051	0.047	0.064	0.066	0.052	0.055	0.052	0.074	0.042	0.070
RSD1 %	2.636	2.558	2.776	2.614	2.429	2.851	3.549	2.702	2.909	2.647	3.482	3.594	3.015	2.591	2.512	3.117	3.060	3.052
RSD2 %	3.111	2.645	3.452	2.859	2.444	2.671	3.219	2.302	2.962	2.755	3.832	3.911	3.096	3.196	2.741	4.487	2.510	4.218
p	0.208	0.741	0.794	0.361	0.772	0.509	0.709	0.364	0.703	0.382	0.853	0.669	0.360	0.133	0.414	0.994	0.562	0.663
r	0.932	0.857	0.820	0.897	0.942	0.833	0.806	0.935	0.895	0.785	0.852	0.833	0.823	0.687	0.871	0.641	0.717	0.601
**DFA**																		
	**O2**	**O1**	**PZ**	**P4**	**P8**	**C4**	**T8**	**P7**	**P3**	**C3**	**FZ**	**F4**	**F8**	**T7**	**F3**	**FP2**	**F7**	**FP1**
AV1	0.416	0.428	0.473	0.456	0.450	0.520	0.573	0.456	0.458	0.518	0.652	0.678	0.740	0.574	0.676	0.837	0.727	0.819
SD1	0.169	0.148	0.177	0.175	0.148	0.149	0.134	0.136	0.154	0.129	0.168	0.147	0.166	0.145	0.188	0.194	0.190	0.198
AV2	0.442	0.445	0.487	0.442	0.467	0.537	0.581	0.457	0.458	0.537	0.615	0.653	0.738	0.566	0.632	0.803	0.724	0.779
SD2	0.218	0.184	0.227	0.172	0.158	0.145	0.132	0.152	0.180	0.153	0.175	0.173	0.179	0.157	0.177	0.198	0.187	0.198

RSD1 %	40.624	34.616	37.436	38.320	32.836	28.729	23.436	29.766	33.598	24.994	25.728	21.672	22.380	25.304	24.611	23.176	26.186	24.236
RSD2 %	49.360	41.449	46.652	38.958	33.899	27.006	22.808	33.308	39.363	28.478	28.377	26.491	24.304	27.651	28.150	24.625	25.765	25.390
p	0.207	0.301	0.607	0.439	0.378	0.415	0.572	0.978	1.000	0.444	0.191	0.356	0.961	0.639	0.140	0.379	0.921	0.257
r	0.944	0.949	0.872	0.918	0.874	0.834	0.913	0.937	0.781	0.772	0.788	0.781	0.818	0.895	0.819	0.687	0.837	0.746
**LZC**																		
	**O2**	**O1**	**PZ**	**P4**	**P8**	**C4**	**T8**	**P7**	**P3**	**C3**	**FZ**	**F4**	**F8**	**T7**	**F3**	**FP2**	**F7**	**FP1**
AV1	0.330	0.332	0.345	0.345	0.343	0.377	0.374	0.349	0.351	0.383	0.355	0.372	0.347	0.368	0.362	0.349	0.343	0.348
SD1	0.038	0.041	0.044	0.044	0.045	0.045	0.060	0.047	0.044	0.041	0.040	0.051	0.035	0.044	0.044	0.035	0.034	0.037
AV2	0.333	0.335	0.338	0.350	0.349	0.391	0.379	0.351	0.349	0.379	0.352	0.365	0.349	0.381	0.363	0.348	0.343	0.352
SD2	0.046	0.043	0.047	0.045	0.041	0.050	0.062	0.045	0.044	0.042	0.042	0.046	0.033	0.061	0.037	0.041	0.028	0.040
RSD1 %	11.669	12.204	12.781	12.630	12.996	12.056	15.942	13.356	12.422	10.594	11.235	13.791	10.074	12.107	12.072	10.065	9.915	10.550
RSD2 %	13.745	12.846	13.841	12.738	11.837	12.891	16.431	12.807	12.738	11.047	11.849	12.499	9.453	16.077	10.167	11.887	8.291	11.286
p	0.587	0.653	0.190	0.171	0.241	0.119	0.615	0.696	0.669	0.334	0.583	0.335	0.658	0.184	0.980	0.989	1.000	0.604
r	0.838	0.865	0.913	0.952	0.888	0.743	0.819	0.920	0.935	0.919	0.882	0.812	0.788	0.751	0.902	0.740	0.753	0.636
